# Outdoor Temperature, Heart Rate and Blood Pressure in Chinese Adults: Effect Modification by Individual Characteristics

**DOI:** 10.1038/srep21003

**Published:** 2016-02-15

**Authors:** Lina Madaniyazi, Yong Zhou, Shanshan Li, Gail Williams, Jouni J.K. Jaakkola, Xin Liang, Yan Liu, Shouling Wu, Yuming Guo

**Affiliations:** 1 School of Public Health and Social Work, Queensland University of Technology, Brisbane, Australia; 2 Department of Neurology, Beijing Tiantan Hospital, Capital Medical University, Beijing, China; 3 Division of Epidemiology and Biostatistics, School of Public Health, University of Queensland, Brisbane, Australia; 4 Center for Environmental and Respiratory Health Research, University of Oulu, Oulu, Finland; 5 Kailuan General Hospital, Tangshan, Hebei Province, China; 6 Department of Cardiology, Kailuan General Hospital, Tangshan, Hebei Province, China

## Abstract

We collected data from Kailuan cohort study from 2006 to 2011 to examine whether short-term effects of ambient temperature on heart rate (HR) and blood pressure (BP) are non-linear or linear, and their potential modifying factors. The HR, BP and individual information, including basic characteristics, life style, socio-economic characteristics and other characteristics, were collected for each participant. Daily mean temperature and relative humidity were collected. A regression model was used to evaluate associations of temperature with HR and BP, with a non-linear function for temperature. We also stratified the analyses in different groups divided by individual characteristics. 47,591 residents were recruited. The relationships of temperature with HR and BP were “V” shaped with thresholds ranging from 22 °C to 28 °C. Both cold and hot effects were observed on HR and BP. The differences of effect estimates were observed among the strata of individual characteristics. The effect estimate of temperature was higher among older people. The cold effect estimate was higher among people with lower Body Mass Index. However, the differences of effect estimates among other groups were inconsistent. These findings suggest both cold and hot temperatures may have short-term impacts on HR and BP. The individual characteristics could modify these relationships.

There is abundant evidence on the association between outdoor temperature and cardiovascular mortality^1^,^2^,^3^. However, it is still not clear what is the mechanism underlying this association. Thus, there is increasing interest in exploring the mechanism behind this association^2^,^3^,^4^,^5^,^6^,^7^, particularly because the extreme weathers will appear frequently under future climate change^8^ and the prevalence of cardiovascular diseases has been increasing^9^. The increased heart rate (HR) and blood pressure (BP) are important cardiac function factors for cardiovascular disease and death^4^. Therefore, the mechanism of temperature-related cardiovascular admission/death may be explained partly by the effects of temperature on HR and BP.

The seasonal variation in HR and BP, including systolic blood pressure (SBP) and diastolic blood pressure (DBP), have been linked to the seasonal changes of outdoor temperature^4^,^5^,^6^,^10^,^11^,^12^,^13^. To date, the long-term effects of temperature on HR and BP have been investigated mostly by cross-sectional and longitudinal studies^4^,^11^,^14^. These studies assumed linear relationships of temperature with HR and BP, and found people living in cold area have higher HR and BP than those living in hot area^4^,^11^,^14^. Very few studies examined the association between short-term exposure of cold temperature and BP, and found cold temperature increased the level of BP^15^. Also, there are very few studies investigating the hot effect of temperature on BP^16^. But no study has examined the impacts of both cold and hot temperatures on BP and HR using the same data. In fact, the association between temperature and cardiovascular mortality has been reported to be non-linear, and both hot and cold temperature increased the risks of cardiovascular mortality^1^,^3^. Therefore, we assumed that a non-linear relationship and an optimum temperature may also exist in the association of temperature with HR and BP.

In China, 45% of all deaths are due to cardiovascular diseases, and the associated health care cost and labour force loss are estimated to excess US$2,500 million per year^7^. However, no study has examined the association of short-term exposure of temperature with HR and BP, although the long-term effect of temperature on BP has been studied by longitudinal studies^14^. In addition, the association of short-term exposure of temperature with HR and BP might be modified by individual characteristics, such as, age, gender and life style^11^,^14^. However, no study has addressed these issues in China.

In this study, we used the Kailuan cohort study to address the following two issues: a) capture the short-term relationships of temperature with HR and BP; b) assess whether the short-term relationships of temperature with HR and BP were modified by individual factors.

## Results

### Data description

Table 1 shows the characteristics of study subjects. The study population of 47,591 subjects included 10,864 female (22.8%) and 36,727 male (77.2%). The mean average age and BMI was 48.1 years and 24.9, respectively. The mean average HR, DBP and SBP were 73.7 beats/min, 81.7 mmHg and 126.3 mmHg, respectively. BP and the incidence of hypertension were higher among the older age group and male subjects. The mean temperature and relative humidity of the study period were 9.9 °C and 64.5%, respectively.

### The associations of temperature with HR and BP in whole study population

**Figure 1:**
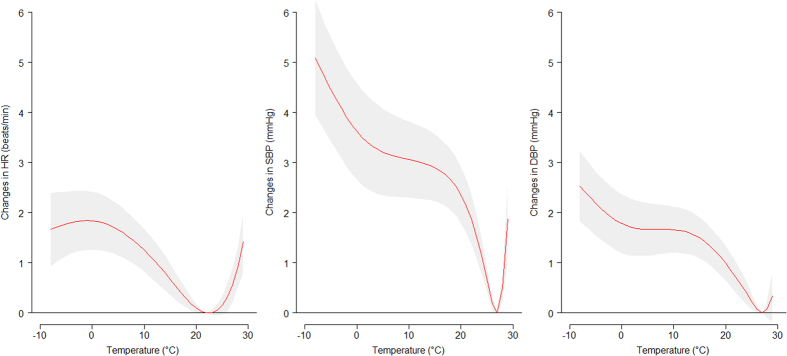
The estimated relationship of 3-day moving average of mean temperature with HR, SBP and DBP among whole study population.

Figure 1 shows the estimated effect of 3-day moving average of mean temperature on HR, SBP and DBP among whole study population. There are V-shaped relationships between mean temperature and HR, SBP and DBP with the most comfortable temperature where the change of HR, SBP and DBP were close to 0. The thresholds (the temperature below which and above which estimates were constrained to be linear) for HR, SBP and DBP were 22 °C, 27 °C and 27 °C, respectively (see Supplementary Table S1 online).

We calculated the cold and hot effect of temperature on HR, SBP and DBP for whole participants (Table 2). For cold effects in whole population, a 1 °C decrease in 3-day moving average of mean temperature below the threshold temperature was associated with 0.063 beats/min (95% confidence interval (CI): 0.039, 0.088) increase in HR, 0.129 mmHg (CI: 0.097, 0.161) increase in SBP and 0.065 mmHg (CI: 0.045, 0.085) increase in DBP. For hot effects in whole population, a 1 °C increase in 3-day moving average of mean temperature above the threshold temperature was associated with 0.133 beats/min (CI: 0.126, 0.204) increase in HR, 0.605 mmHg (CI: 0.175, 1.036) increase in SBP and 0.128 mmHg (CI: −0.135, 0.391) increase in DBP.

### The associations of temperature with HR and BP in different groups

We stratified the study population by different characteristics, including basic characteristics, life style, socio-economic characteristics and other characteristics. Then we estimated the associations of temperature with HR, SBP and DBP for each group. We only reported the effect estimates for those groups with clear V-shaped associations (Table 2). For those groups without clear thresholds, we did not examine the effects of cold and hot temperature on HR, SBP and DBP.

In general, the associations of temperature with HR, SBP and DBP were V-shaped in all groups (see Supplementary Figs S1–S6), except for the following groups: people older than 65 years old, female, people who smoke often, people with higher income, intellectual workers, and people with hyperlipidaemia.

Except for cold effect estimate on HR, the effect estimates on HR, SBP and DBP were higher among people aged among 45–64 years old than younger people (“<44” age) (Table 2). However, only the differences of hot effect estimates on BP between two age groups were statistically significant. The cold effect estimates on HR and BP were highest among people with low BMI (<18.5) (Table 2), but the differences were not statistically significant. The differences of effects estimates between other sub-groups were inconsistent.

## Discussion

Generally, the temperature-HR, SBP and DBP relationships in Kailuan were V-shaped, with thresholds where the change of HR, SBP and DBP were close to 0. However, the differences of effect estimates between sub-groups (e.g., the difference of estimated hot effect between male and female) were inconsistent.

### The associations of temperature with HR and BP

In general, the temperature-HR and temperature-BP relationships were observed to be V-shaped in most groups. Similarly, a non-linear relationship was reported between temperature and heart rate variability among aging population during warm season in Boston^17^. However, most studies explored the long-term effect of temperature on HR and BP^4^,^11^,^14^, with quite few studies on its short-term effect^15^,^16^. In addition, all these studies assumed a linear relationship of temperature with HR and BP^4^,^11^,^14^,^15^,^16^. In China, effects of temperature on BP have been estimated by longitudinal studies^14^. To our best knowledge, no study has analysed short-term effects of temperature on HR and BP in China yet. However, there are a lot of studies addressing the relationships between temperature and cardiovascular mortality in China^3^,^18^,^19^,^20^,^21^,^22^. Thus, we compared our results with these studies. A nonlinear relationship between temperature and cardiovascular mortality was evident across different studies that reported U-, V-, J-shaped patterns^3^,^18^,^19^,^20^,^21^,^22^, which is similar to our findings of V-shaped relations between temperature-HR, SBP and DBP. Therefore, our results may explain the temperature-cardiovascular mortality relationships. In addition, temperature thresholds have been reported by most of these studies (see Supplementary Table S2)^3^,^18^,^19^,^20^,^21^. For example, Huang *et al.* found a U-shaped relationship between temperature and cardiovascular mortality in Changsha, with a threshold ranging from 10 °C to 29 °C^18^. Similarly, Guo *et al.*^3^ found both cold and hot temperatures were associated with cardiovascular mortality in Tianjin, which is close to Kailuan, with a large range of temperature from 0.6 °C to 25.1 °C. In our study, instead of such a large temperature range for thresholds, we found an optimum temperature in whole study subjects: 22 °C for HR, 27 °C for SBP and 27 °C for DBP, respectively (see Supplementary Table S1).

However, the relationships of temperature with HR and BP were not “V” shaped in the following groups: a) people aged over 65 years; b) female; c) intellectual workers; and d) people who smoke often; e) people with hyperlipidaemia. In general, in lower and higher tails of temperature distribution, a positive temperature-HR relationship or a negative temperature-BP relationship has been found in these groups, except for the “V” shaped relationships of temperature with HR among people who smoke often, as well as the “V” and “J” shaped relationships of temperature with SBP and DBP among people with hyperlipidaemia. The negative relationship between temperature and BP among people aged over 65 years and female might be resulted from people’s protective activities. For example, most of the older people were retired from work, and they intend to stay outdoors when it’s warmer while prefer to stay indoors when it’s cold or hot. The female prefers to stay indoors or use umbrellas outside when it’s hot. The difference between temperature-HR relationship and temperature-BP relationship among people aged over 65 years and female indicates that increased BP resulting from cold and hot effect may be avoided by people’s protective activities. For intellectual workers, people who smoke often, and people with hyperlipidaemia, the relationships of temperature with HR and BP were difficult to be concluded. However, these findings were intriguing and suggestive indications for modifying effect of the baseline characteristics on the relationships of temperature with HR and BP.

### The association estimates on HR and BP

Many studies have examined the effects of temperature on HR and BP by cross-sectional and longitudinal studies worldwide^4^,^5^,^6^,^10^,^11^,^12^,^13^,^14^. An increase in BP associated with exposure to decreasing outdoor temperature was reported by these studies^4^,^5^,^6^,^10^,^11^,^12^,^13^,^14^. The effects of increasing outdoor temperature on HR and BP only has been reported by one study: SBP was found to decrease with the increase in outdoor temperature^16^. However, in our study, both cold and hot effects were discovered: the increased HR and BP were found with the decrease/increase in temperature below/above the threshold. The reason for this inconsistence might be the linear temperature-HR and BP relationship assumed by these studies, whereas we assumed non-linear relationships of temperature-HR and BP. Assuming linear relationships may be unable to capture the real relation, and thus underestimate the hot effect.

### The modifying effects of individual characteristics

We also analysed the modifying effects of baseline characteristics on the relationships of temperature with HR and BP. In general, effect estimates of temperature were higher among people aged between 45 and 64 years old than people younger than 45 years old; the cold effect estimates were higher among people with lower BMI (<18.5). Likewise, higher effects of temperature on cardiovascular mortality have been reported among older people^23^,^24^. In addition, seasonal variation in BP was reported to be larger among elderly individuals^25^,^26^. One possible explanation might be that the autonomic response to cold/ hot decreases with aging^25^,^27^,^28^. Thus, our result indicates that older individuals may be much more susceptible to cold and heat stress than younger people.

The higher cold effect was observed among people with low BMI (<18.5). One possible reason may be that more body fat could provide better insulation, and hence leads to smaller changes in BP with exposure to low temperature. In contrast, other studies reported greater associations between temperature and BP among obese subjects^6^,^10^. These two studies focused specifically on older individuals, while we investigated the whole population^6^,^10^. It might be the possible reason for the inconsistent findings on the modifying effect of BMI on the associations of temperature and BP. On the other hand, it also indicates that the modifying effect of BMI may differ in different age groups.

The differences of effect estimates among other groups were inconsistent. Similarly, results from other studies were also inconsistent. Kent *et al.* found that inverse relationships between temperature and BP had negligible differences by education, income, sex and age^11^. However, other studies, which discovered cold effect of temperature on BP, reported controversial results on the potential modifying effects of baseline characteristics on temperature-BP relationships^5^,^13^,^14^,^29^. For example, Chen *et al.* found cold effect was higher on male than on female^14^, while Barnett *et al.* discovered it was higher on female than on male^13^. These controversial results may result from the following two possible reasons: a) the relationship between baseline characteristics and BP may not be linear. For example, U-shaped relationship has been reported between alcohol consumption and BP^29^; b) These baseline characteristics may have interactive effect on BP. For example, it has been discovered that smoking-BP relationship could be affected by alcohol intake and BMI^29^. However, our finding was intriguing and suggestive indication for some modifying effect of baseline characteristics on the effect of temperature on HR and BP.

### Strengths and limitations

This is the first study to assess the non-linear relationships of temperature with HR and BP, and its possible modifying factors in China. First, we examined the cold and hot effect of temperature on HR, SBP and DBP and explored corresponding thresholds. Then, we examined the modifying effect of 12 individual characteristics on the effects of temperature on HR and BP. Our findings cannot only help explore the possible mechanism under the temperature-cardiovascular mortality relationship but also assist public and government in developing better local response for extreme temperature.

One limitation of our study is that the data on temperature was obtained from fixed sites rather than individual exposure, which might cause some measurement error. However, because temperatures are highly correlated within the city, we assume the results could not be biased^30^. Our results were based on the data from only one city, which is difficult to generalize them to other regions. In addition, the confounding effect of air pollutants was not considered in our study, because the data were unavailable. The interactive effects of individual characteristics were not included in our study, though it has been reported by quite a few studies^29^. Further study needs to be conducted to address these issues.

## Conclusion

Our findings suggest both extreme cold and hot temperatures have short-term impacts on heart rate and BP. The individual characteristics could modify these relationships.

## Method

### Study population

The present analyses are based on data collected in the Kailuan Study. The study was carried out in the Kailuan community in Tangshan City, which is a large and littoral modern city located in the central area of the circulating Bohai Sea Gulf region. This study has been described in details elsewhere^31^. The Kailuan cohort study was a prospective community-based cohort study aiming to investigate risk factors for chronic diseases (such as stroke, myocardial infarction, cancers, etc.). This study provided an opportunity to investigate the short-term relationships between outdoor temperature and HR and BP among those participants.

In brief, all residents in Kailuan community have routine health examination in 11 hospitals every two years, supported by Kailuan Group which is a coal mining company. 101,510 residents (81,110 men and 20,400 women, aged 18–98 years old) who had routine health examination from 2006 to 2007 were enrolled and followed up until 2011 in Kailuan study. The data collection included a health examination and a face-to-face questionnaire. The health examination includes physical examination, routine blood, urine, and biochemical tests. The questionnaire were taken at every two-year health examination and collected information on basic characteristics (age, gender and Body Mass Index (BMI)), life style (smoking status, drinking habit, physical activities and salt intake), socio-economic characteristics (income, work type and education levels) and other characteristics (hypertension, hyperlipidaemia, medical history, the existence of pre-existing stroke or myocardial infarction (MI), hypertension history of mother, and hypertension history of father). In the current study, in order to avoid the modifying effect of antihypertensive medications on the associations of outdoor temperature with HR and BP, 47,591 residents who did not use any antihypertensive medications and did not have any missing values were included in our study.

The study was approved by the University of Queensland Medical Research Ethics Committee. The methods were carried out in accordance with the approved guidelines. The informed consent from all subjects participating in the study was received prior to conducting the study.

### Health measurement

At each visiting of health examination, resting HR, SBP and DBP were measured in duplicate in sitting position after 3 minutes of rest. SBP and DBP were measured by a mercury sphygmomanometer at 5-minute intervals. The average of the two readings was used for the current data analyses. If the difference between the two measurements exceeded 5 mm Hg, an additional reading was taken, and the average of the three readings was used. The date (year, month and day) of the health examination was recorded for each participant.

### Potential modifying factors and meteorologic factors

Individual Information of each participant, including age, sex, body mass index (BMI), smoking habit, drinking habit, physical activity, salt intake, income, education level, work type (intellectual/manual), hypertension (yes/no), hyperlipidaemia (yes/no), the existence of pre-existing stroke or MI (yes/no), hypertension history of mother (yes/no), and hypertension history of father (yes/no) was collected by the face-to-face questionnaires by well-trained research doctors and nurses.

The age was classified as “<44”, “45–64” and “≥65”; the BMI was also classified as “<18.5”, “18.5–23.9” and “≥23.9”. According to the self-reported information, the smoking habit was classified as “never or former”, “occasionally”, or “often”. The drinking habit was classified as “never or former or occasionally”, or “often”. Salt intake was classified as “low or medium”, or “high”. Physical activity was evaluated based on the responses to questions regarding the type and frequency of physical activity at work and during leisure time. Physical activity was classified as “inactive (<80 minutes per week)” or “active (>4 times per week and >20 minutes at a time)”. For socio-economic characteristics, the average monthly income of each participant was categorized as “<¥800” and “≥¥800”; the education level was categorized as “elementary or below” and “middle school or above”; the work type was categorized as “intellectual” and “manual”.

Hypertension was defined as having a history of hypertension, or a SBP≥140 mmHg, or a DPB ≥90 mmHg, or using antihypertensive medications. The existence of pre-existing stroke or MI, and hypertension history of mother and father was defined as any self-reported previous physician diagnosis of stroke or MI. The use of antihypertensive medications within the past 2 weeks of the baseline interview was self-reported.

Daily mean temperature and relative humidity for the study period were collected from Tangshan Meteorological Bureau during the study period. The monitoring station is 1 km away from the study community.

### Statistical analysis

We used a regression model to examine the effect of temperature on HR and BP. The potential nonlinear effects of temperature on HR and BP were modelled by a natural cubic spline with three degrees of freedom (*df*) for temperature. The seasonal and long-term trends of HR and BP were controlled by categorical variables for the year and calendar month. Relative humidity, age and BMI were controlled using a cubic spline with four *df*. Other modifying factors were adjusted as categorical variables, including drinking habit, education level, gender, history of myocardial infarction, hypertension, hypertension history of parent, hyperlipidaemia, income, marriage status, physical activities, salt intake, smoking status, and work type. To control for the measurement bias by hospital, we included hospital as a categorical variable in the model.

Single-day lag models might underestimate the cumulative effect of temperature on HR and BP. According to a previous study^6^, we used a 3-day moving average of current and previous 2 day’s (lag 0–2 days) of temperature in our analysis. In addition, our initial analysis found that the associations of temperature with HR and BP were V-shaped, with a potential threshold. Therefore, we examined the threshold by a segment spline, assuming the effect of temperature is linear below or above the threshold. The threshold was identified with the lowest HR and BP. Then a linear function below/above threshold was used to calculate the change in HR and BP for a 1 °C of temperature change.

We assessed the associations of temperature with HR and BP for all participants. We also stratified the analyses in different groups divided by individual characteristics.

The statistical significance of difference between effect estimates of the modification factors (e.g., the difference among different age groups), was examined by


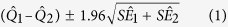


where 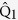 and 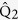 are the estimates for the two categories, and *S*Ê_1_ and *S*Ê_2_ are their respective SEs^2^,^32^. Regardless of significance, we considered modification of effect by a factor of ≥2 to be important and worthy of attention^2^,^32^.

The model fitness was assessed by Akaike information criterion (AIC). Sensitive analyses were also performed by changing model specifications, for example, *df* for temperature, relative humidity, and age. R software (version 3.0.2) was used in our analysis.

## Additional Information

**How to cite this article**: Madaniyazi, L. *et al.* Outdoor Temperature, Heart Rate and Blood Pressure in Chinese Adults: Effect Modification by Individual Characteristics. *Sci. Rep.* **6**, 21003; doi: 10.1038/srep21003 (2016).

## Supplementary Material

Supplementary Information

## Figures and Tables

**Table 1:** The Summary of Baseline Characteristics and Meteorology of Study Area.

Basic Characteristics		Whole				Age Groups				Gender								
				Age 1(≥44)		Age 2 (45–65)		Age 3 (>65)		Female		Male						
Heart rate, mean (SD), beats/min		73.3	9.9	74.0	9.9	73.0	9.8	72.3	10.7	73.2	9.4	10.0						
Blood Press, mean (SD), mmHg																		
Diastolic		81.7	10.9	79.7	10.8	82.9	10.9	82.2	10.2	77.8	10.1	82.8	10.8					
Systolic		126.3	18.6	120.5	16.2	128.7	18.7	136.9	20.1	120.4	18.4	120.0	18.3					
Age, mean (SD), y		48.1	11.6	N.A.		N.A.		N.A.		46.7	10.8	48.5	11.8					
BMI		24.9	3.4	24.7	3.7	25.0	3.3	24.5	3.4	24.3	3.7	25.1	3.4					
Gender, No. (%)																		
Female		10,864	22.8	5,005	25.9	5.247	21.6	612	15.3	N.A.								
Male		36,727	77.2	14,306	74.1	19,032	78.4	3,389	84.7	N.A.								
Life Style	Smoking habit, No. (%)																	
	Never or former	31,105	65.5	12,197	63.2	2,683	65.6	3,025	76.1	10,699	98.6	20,406	55.7					
	Occasionally	1,854	3.9	1,097	5.7	670	2.8	87	2.2	50	0.5	1,804	4.9					
	Often	14,528	30.6	6,004	31.1	7,662	31.6	862	21.7	101	0.9	14,427	39.4					
	Drinking habit, No. (%)																	
	Never/used to/occasionally	39,173	82.5	16,429	85.1	19,418	80.2	3,326	83.6	10,794	99.5	28,379	77.4					
	Often	8,331	17.5	2,873	14.9	4,806	19.8	652	16.4	54	0.5	8,277	22.6					
	Salt Consumption, No. (%)																	
	Low or Medium	42,783	90.1	17,372	90.0	21,797	90.0	3,614	90.9	10,150	93.6	32,633	89.0					
	High	4,716	9.9	1,931	10.0	2,424	10.0	361	9.1	691	6.4	4,025	11.0					
	Physical activities, No. (%)																	
	Inactive	41,326	87.1	18,004	93.3	20,538	84.9	2,784	70.2	9,586	88.5	31,740	86.7					
	Active	6,131	12.9	1,289	6.7	3,660	15.1	1,182	29.8	1,251	11.5	4,880	13.3					
Socio-economic Characteristics	Education level, No. (%)																	
	Elementary or below	3,259	6.9	349	1.8	1,908	7.9	1,002	25.1	393	3.6	2,866	7.8					
	Middle school or above	44,299	93.2	18,949	98.2	22,357	92.1	2,993	74.9	10,467	96.4	33,832	92.2					
	Income, No. (%)																	
	<¥800	40,541	85.3	16,144	83.7	21,035	86.8	3,362	84.5	9,190	84.7	31,351	85.5					
	≥¥800	6,982	14.7	3,154	16.3	3,210	13.2	628	15.5	1,662	15.3	5,320	14.5					
	Work type, No. (%)																	
	Intellectual	3,738	7.9	1,741	9.0	1,480	6.1	517	13.0	1,432	13.2	2,306	6.3					
	Manual	43,793	92.1	17,561	91.0	22,765	93.9	3,467	87.0	9,413	86.8	34,380	93.7					
Other Characteristics	Hypertension, No. (%)																	
	No	32,339	68.0	14,890	77.1	15,403	63.4	2,046	51.1	8,587	79.0	23,752	64.7					
	Yes	15,252	32.1	4,421	22.9	8,876	36.6	1,955	48.9	2,277	21.0	12,975	35.3					
	Hyperlipidemia No. (%)																	
	None	45,935	96.5	19,000	98.4	23,200	95.6	3,735	93.4	10,407	95.8	35,528	96.7					
	Yes	1,655	3.5	311	1.6	1,079	4.4	265	6.6	457	4.2	1,198	3.3					
	Hypertension history of father, No. (%)																	
	No	43,693	91.8	17,285	89.5	22,540	92.8	3,868	96.7	90,807	90.3	33,886	92.3					
	Yes	3,898	8.2	2,026	10.5	1,739	7.2	133	3.3	1,057	9.7	2,841	7.7					
	Hypertension history of mother, No. (%)																	
	No	43,551	91.5	17,213	89.1	22,440	92.4	3,898	97.4	9,742	89.7	33,809	92.1					
	Yes	4,040	8.5	2098	10.9	1,839	7.6	103	2.6	1,122	10.3	2,218	7.9					
	History of myocardial infarction, No. (%)																	
	No	47,279	99.3	19,258	99.9	24,073	99.2	3,921	98.0	10,831	99.7	36,448	99.3					
	Yes	308	0.7	25	0.1	203	0.8	80	2.0	32	0.3	276	0.8					
	Marriage status, No. (%)																	
	Single	879	1.9	823	4.3	52	0.2	4	0.1	156	1.4	723	2.0					
	Married	45,265	95.2	18,090	93.7	23,552	97.0	3,623	90.7	10,216	94.1	35,049	95.5					
	Divorce	421	0.9	217	1.1	185	0.8	19	0.5	164	1.5	257	0.7					
	Widowed	572	1.2	59	0.3	254	1.1	259	6.5	236	2.2	336	0.9					
	Encore	437	0.9	120	0.6	229	0.9	88	2.2	89	0.8	348	1.0					

**Table 2:** The Cold and Hot Effects of 3-day Moving Average of Mean Temperature on HR, SBP and DBP.

Modifying Characteristics		HR (beats/min)		SBP (mmHg)		DBP (mmHg)	
		Cold Effect^a^	Hot Effect^b^	Cold Effect^a^	Hot Effect^b^	Cold Effect^a^	Hot Effect^b^
Basic Characteristics	Whole	0.063* (0.031, 0.088)	0.133^b^ (0.063, 0.204)	0.129^b^ (0.097, 0.161)	0.605^*^ (0.175, 1.036)	0.065^*^ (0.045, 0.085)	0.128 (−0.135, 0.391)
	Age						
	<44	0.119* (0.078,0.161)	0.177* (0.040, 0.314)	0.071* (0.014, 0.128)	− 0.028 (−0.242, 0.187)	0.040* (0.001, 0.078)	0.038 (−0.104, 0.181)
	45–64	0.051*(0.021,0.082)	0.314* (0.167, 0.461)	0.173* (0.123, 0.223)	0.707* (0.039, 1.376)	0.082* (0.052, 0.111)	0.110 (−0.285, 0.505)
	≥ 65	N.A.	N.A.	N.A.	N.A.	N.A.	N.A.
	Gender						
	Male	0.084* (0.056,0.112)	0.178* (0.089, 0.267)	0.130* (0.088, 0.172)	0.636 (0.111, 1.160) *	0.072* (0.046, 0.097)	0.170 (−0.148, 0.489)
	Female	N.A.	N.A.	N.A.	N.A.	N.A.	N.A.
	BMI						
	<18.5	0.302* (0.062,0.543)	0.158 (−0.333, 0.650)	0.379* (0.089, 0.668)	1.099 (−9.186, 11.381)	0.155 (−0.008, 0.138)	-0.148 (−2.581, 2.285)
	18.5–23.9	0.079^*^ (0.038,0.119)	0.238^*^ (0.125, 0.351)	0.103^*^ (0.046, 0.160)	0.527 (−0.221, 1.275)	0.062^*^ (0.028, 0.097)	0.332 (−0.117, 0.781)
	≥23.9	0.045* (0.016,0.075)	0.089 (−0.028, 0.207)	0.133* (0.086, 0.181)	0.866* (0.226, 1.507)	0.061* (0.033, 0.090)	0.112 (−0.274, 0.498)
Life Style	Smoking habit						
	Never or former	0.020 (−0.010, 0.049)	0.124 (−0.112, 0.360)	0.146* (0.099, 0.093)	0.408 (−0.280, 1.097)	0.073 (−0.046, 0.101)	0.236 (−0.171, 0.643)*
	Occasionally	0.111* (0.035, 0.187)	0.221* (0.050, 0.393)	0.120 (−0.021, 0.220)	0.282 (−0.197, 0.760)	0.097 (0.034, 0.159)	0.082 (−0.220, 0.384)
	Often	0.116* (0.067, 0.166)	0.297* (0.107, 0.487)	N.A.	N.A.	N.A.	N.A.
	Drinking Status						
	Never/former/occasionally	0.034*(0.009, 0.060)	0.126* (0.029, 0.223)	0.121* (0.089, 0.159)	0.660* (0.221, 0.114)	0.059* (0.037, 0.080)	0.239 (−0.034, 0.512)
	Often	0.121* (0.057, 0.185)	0.443* (0.190, 0.695)	0.086 (−0.007, 0.178)	0.087 (−0.461, 0.636)	0.060 (−0.010, 0.129)	0.080 (−0.025, 0.186)
	Salt intake						
	Low or medium	0.036* (0.011, 0.062)	0.157* (0.058, 0.255)	0.121* (0.081, 0.160)	0.660* (0.120, 1.201)	0.056* (0.032, 0.079)	0.309 (−0.016, 0.634)
	High	0.121* (0.050, 0.193)	0.237 (−0.079, 0.552)	0.139* (0.027, 0.250)	0.250 (−1.015, 1.515)	0.050 (−0.017, 0.117)	-0.527 (−2.151, 1.097)
	Physical Activity						
	Inactive	0.051* (0.024,0.077)	0.118* (0.030, 0.206)	0.110* (0.070, 0.150)	0.614* (0.089, 1.139)	0.048* (0.024, 0.072)	0.172 (−0.147, 0.490)
	Active	0.045 (−0.018,0.109)	0.203 (−0.019, 0.426)	0.169* (0.068, 0.269)	0.301 (−1.218, 1.819)	0.089* (0.031, 0.148)	0.214 (−0.245, 0.673)
Socio-economic characteristics	Education level						
	Elementary or below	0.002 (−0.103,0.106)	0.300 (−0.100, 0.700)	0.255* (0.082, 0.427)	0.469 (−0.423, 1.362)	0.176* (0.077, 0.274)	0.314 (−0.063, 0.691)
	Middle school or above	0.048* (0.023,0.073)	0.141* (0.047, 0.235)	0.113* (0.075, 0.151)	0.596* (0.089, 1.104)	0.048* (0.025, 0.071)	0.200 (−0.106, 0.507)
	Income						
	<¥800	0.051* (0.024, 0.079)	0.200* (0.089, 0.311)	0.143* (0.099, 0.187)	1.043* (0.348, 1.739)	0.066* (0.039, 0.092)	0.556* (0.136, 0.976)
	≥¥800	N.A.	N.A.	N.A.	N.A.	N.A.	N.A.
	Work Type						
	Intellectual	N.A.	N.A.	N.A.	N.A.	N.A.	N.A.
	Manual	0.048* (0.024,0.073)	0.137* (0.041, 0.232)	0.141* (0.102, 0.180)	0.651* (0.136, 1.166)	0.065* (0.042, 0.089)	0.225 (−0.085, 0.534)
Other Characteristics	Hypertension						
	No	0.051* (0.024, 0.079)	0.232* (0.123, 0.341)	0.116* (0.081, 0.151)	0.652* (0.166, 1.138)	0.038* (0.016, 0.060)	0.002 (−0.302, 0.306)
	Yes	0.035 (−0.010, 0.081)	0.066 (−0.144, 0.276)	0.115* (0.048, 0.182)	0.185 (−0.659, 1.029)	0.066* (0.026, 0.106)	0.406 (−0.093, 0.906)
	Hyperlipidemia						
	No	0.050*> (0.026, 0.074)	0.144* (0.052, 0.237)	0.125* (0.091, 0.158)	0.607* (0.160, 1.054)	0.056* (0.035, 0.076)	0.218 (−0.056, 0.492)
	Yes	N.A.	N.A.	0.065 (−0.118, 0.248)	0.520 (−0.425, 1.466)	0.014 (−0.094, 0.121)	N.A.

^a^The increase in HR (beats/min), SBP (mmHg) and DBP (mmHg) for a 1 °C of temperature decrease below the thresholds (see Supplementary Table S1 online).

^b^The increase in HR (beats/min), SBP (mmHg) and DBP (mmHg) for a 1 °C of temperature increase above the thresholds (see Supplementary Table S1 online); *p < 0.05.
