# Supplementary material for: Outdoor Temperature, Heart Rate and Blood Pressure in Chinese Adults: Effect Modification by Individual Characteristics
Source: Sci Rep. 2016 Feb 15;6:21003. doi: 10.1038/srep21003 (PMC4753475; doi:10.1038/srep21003)
Supplement: Supplementary Information [file srep21003-s1.pdf]

Supplemental Material

Outdoor Temperature, Heart Rate and Blood Pressure in Chinese Adults: Effect Modification by Individual Characteristics

Table S1. Thresholds(°C) for effects of temperature on HR, SBP and DBP

	HR	SBP	DBP
Whole	22	27	27
Age			
<44	23	24	24
45-64	25	27	27
≥ 65	N.A.	N.A.	N.A.
Gender			
Male	N.A.	N.A.	N.A.
Female	23	27	27
BMI			
<18.5	20	28	27
18.5-23.9	22	27	27
≥23.9	24	27	27
Education level			
Elementary or below	23	25	23
Middle school or above	24	27	27
Work Type			
Mental			
Manual	24	27	27
Physical Activity			
Inactive	23	27	27
Active	23	27	26
Income			
<¥800	24	27	27
≥¥800			
Smoking status			
Never or former	26	27	27
Current but occasionally	23	26	26
Current and often			
Salt Intake			
Low or medium	24	27	27
High	25	27	28
Drinking status			
Never/former/current but occasionally	24	27	27
Current and often	23	25	15
Hypertension			
No	24	27	27
Yes	25	27	27
Hyperlipidemia			
No	24	27	27
Yes		25	25

Table S2. The comparison of the thresholds of associations with cardiovascular systems (cardiovascular/cerebrovascular mortality, HR, SBP and DBP) in different Chinese cities

City	Threshold (°C)			
	Temperature-Cardiovascular Mortality Associations		Temperature-Cerebrovascular Mortality Associations	Temperature-HR/SBP/DBP Associations
Our study				22/ 27/ 27
Harbin [1]	20.6			
Changchun [1]	20.6			
Shenyang [1]	21.5			
Beijing [1, 2]	24.9		0.7 †	
Tianjin [1-3]	24.5	0.6-25.1 *	-0.6 †	
Shijiazhuang [1]	23.8			
Jinan [1]	24.9			
Zhengzhou [1]	25.9			
Shanghai [1,2]	24.5		26.9 †	
Nanjing [1]	27.9			
Suzhou [4]	26			
Chengdu [1]	24.1			
Chongqing [1]	29.2			
Changsha [1, 5]	25.1	10-29 ‡		
Wuhan [2]			25 †	
Kunming [1]	23.3			
Guangzhou [1,2]	29.0		27.8 †	

** The result was obtained from the 3rd literature. † The result was obtained from the 2nd literature.*
‡ The result was obtained from 5th literature.

Abbreviations: HR: Heart Rate; SBP: Systolic Blood Pressure; DBP: Diastolic Blood Pressure

Reference:

1. Yang, J., Yin, P., Zhou, M. et al. Cardiovascular mortality risk attributable to ambient temperature in China. Heart, doi:10.1136/heartjnl-2015-308062 (2015).
2. Zhang, Y., Li, S., Pan, X. et al. The effects of ambient temperature on cerebrovascular mortality: An epidemiologic study in four climatic zones in China. Environmental Health 13, 24 (2014).
3. Guo, Y., Barnett, A. G., Pan, X. et al. The impact of temperature on mortality in Tianjin, China: A case−crossover design with a distributed lag non-linear model. Environ. Health Perspect. 119, 1719-1725 (2011).
4. Wang, C., Chen, R., Kuang, X. et al. Temperature and daily mortality in Suzhou, China: A time series analysis. Sci. Total Environ. 466-467, 985-990, doi:10.1016/j.scitotenv.2013.08.011 (2014).
5. Huang, J., Wang, J. & Yu, W. The lag effects and vulnerabilities of temperature effects on cardiovascular disease mortality in a subtropical climate zone in China. Intl. J. Environ. Res. Publ. Health 11, 3982-3994 (2014).

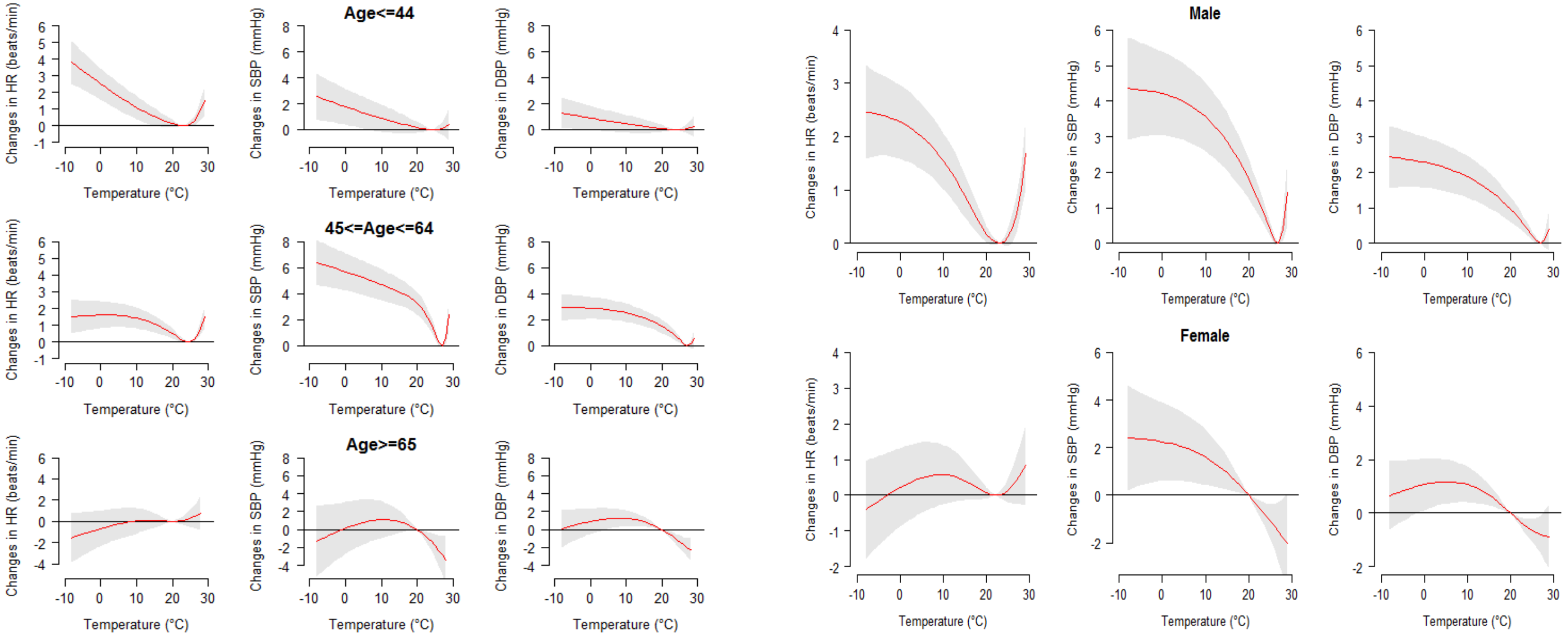

(A) (B)

Figure S1. The estimated effects of 3-day moving average of mean temperature on HR, SBP and DBP among age groups (A) and gender (B)

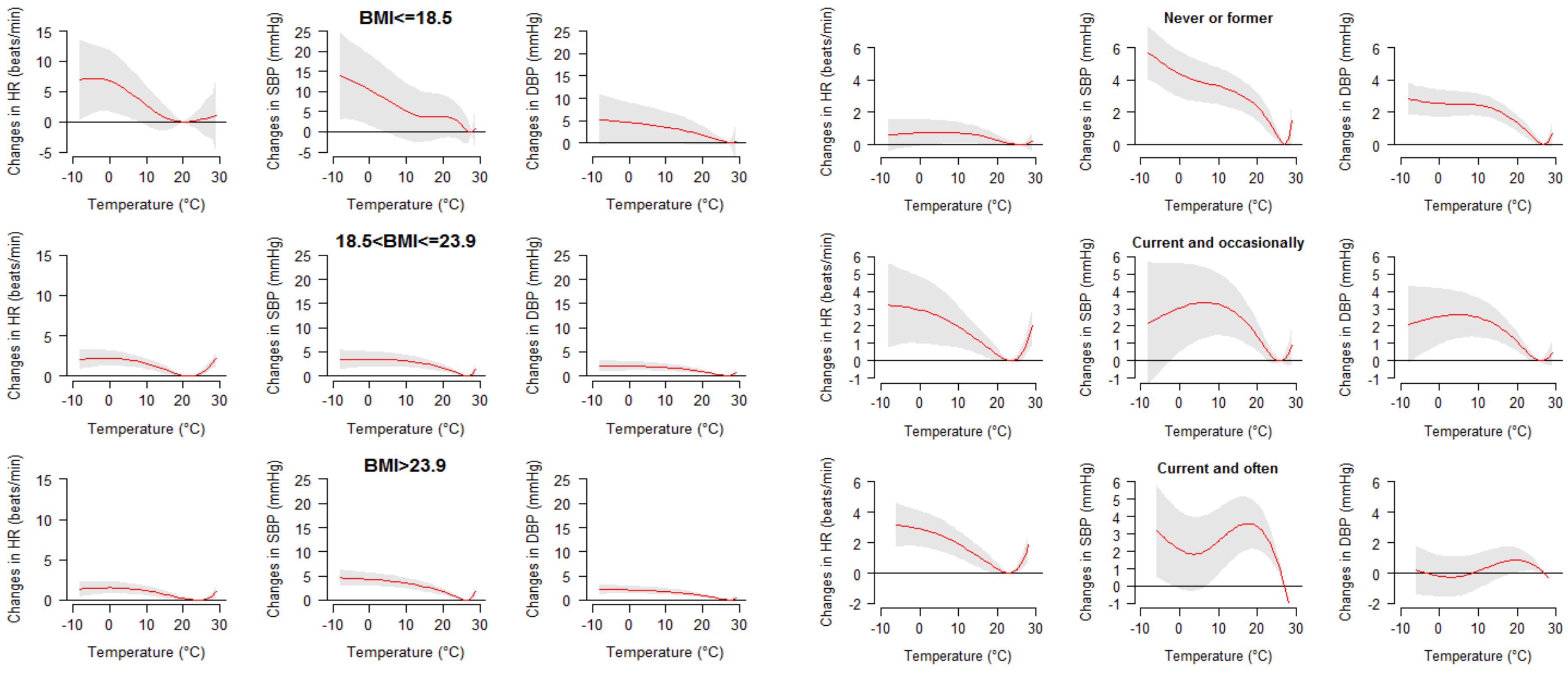

(A)

(B)

Figure S2. The estimated effects of 3-day moving average of mean temperature on HR, SBP and DBP among BMI groups (A) and people with different smoking habits (B)

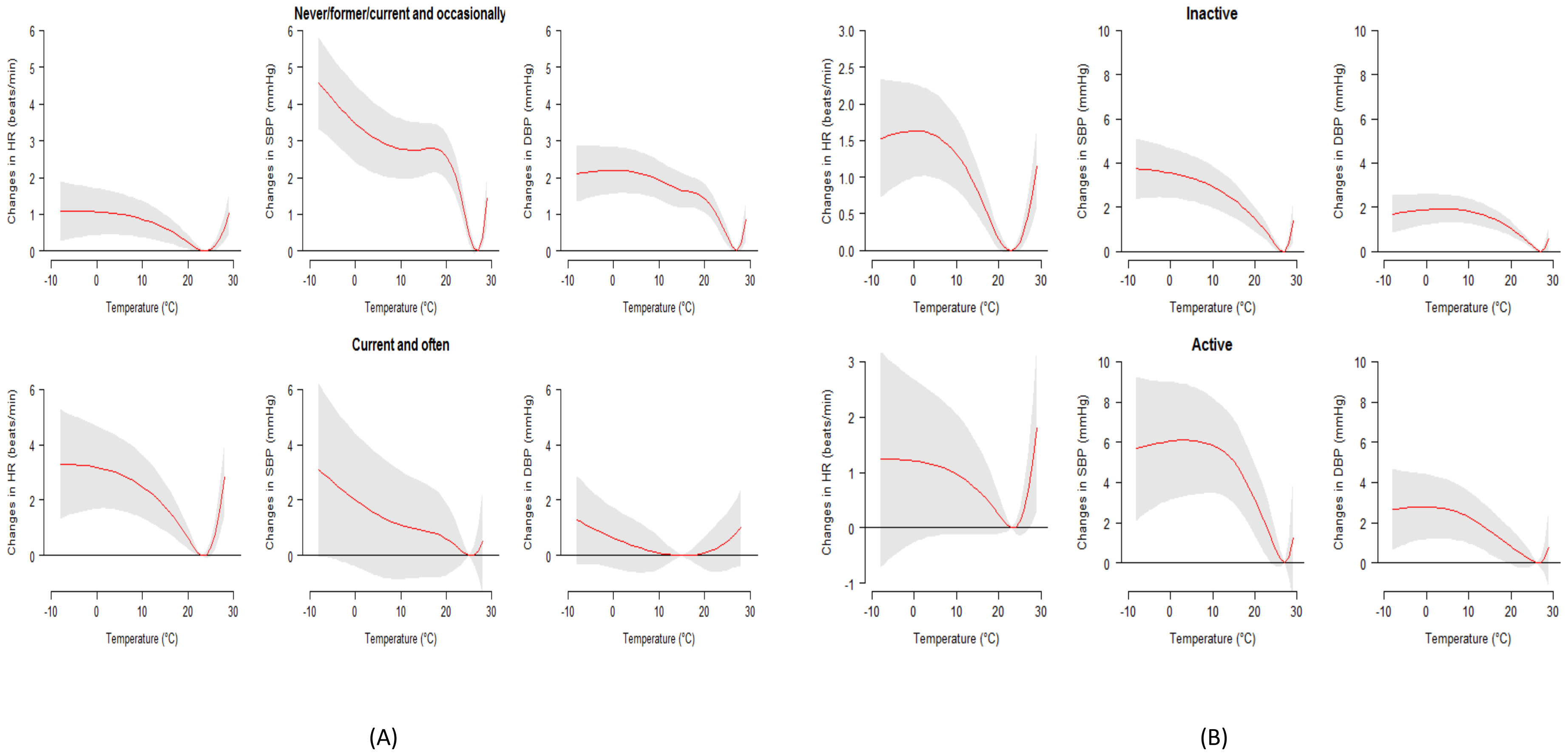

Figure S3. The estimated effects of 3-day moving average of mean temperature on HR, SBP and DBP among people with different drinking habits (A) and different physical activities (B)

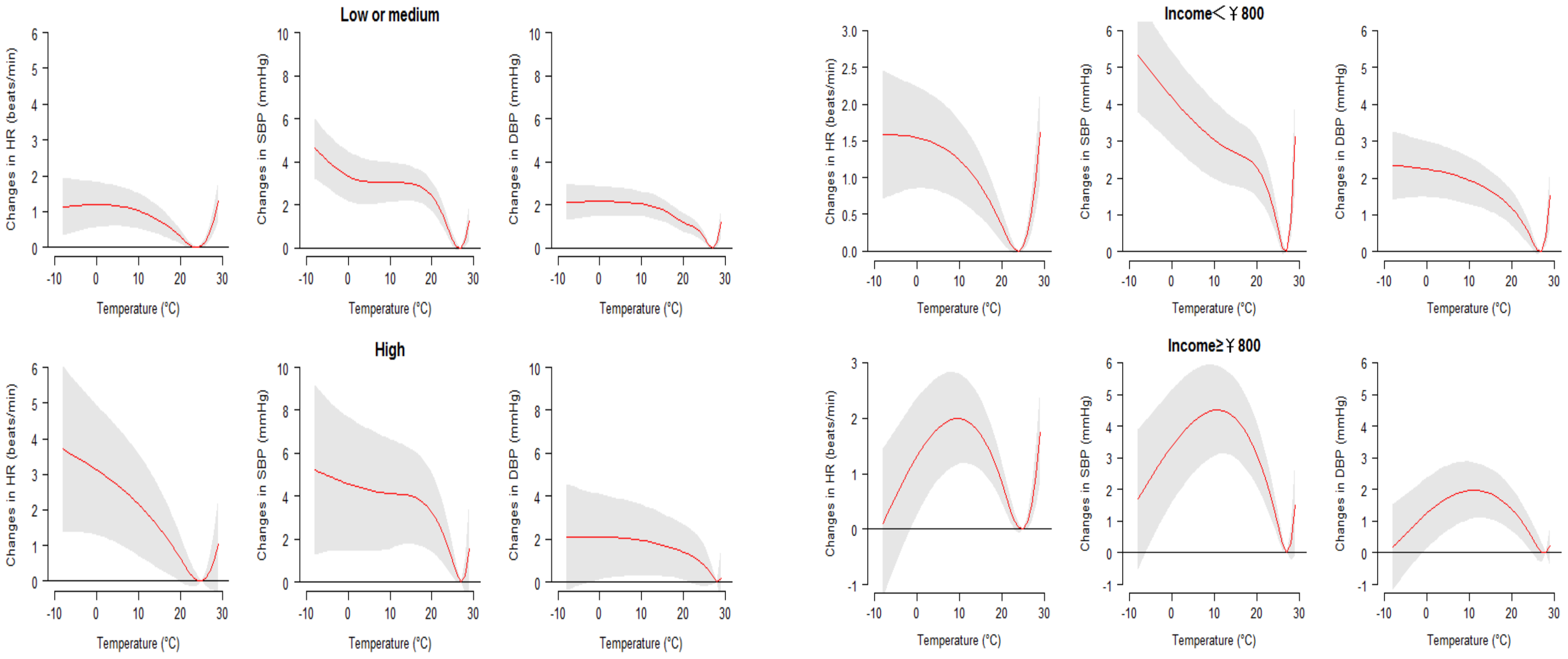

(A) (B)

Figure S4. The estimated effects of 3-day moving average of mean temperature on HR, SBP and DBP among people with different salt intake (A) and different income (B)

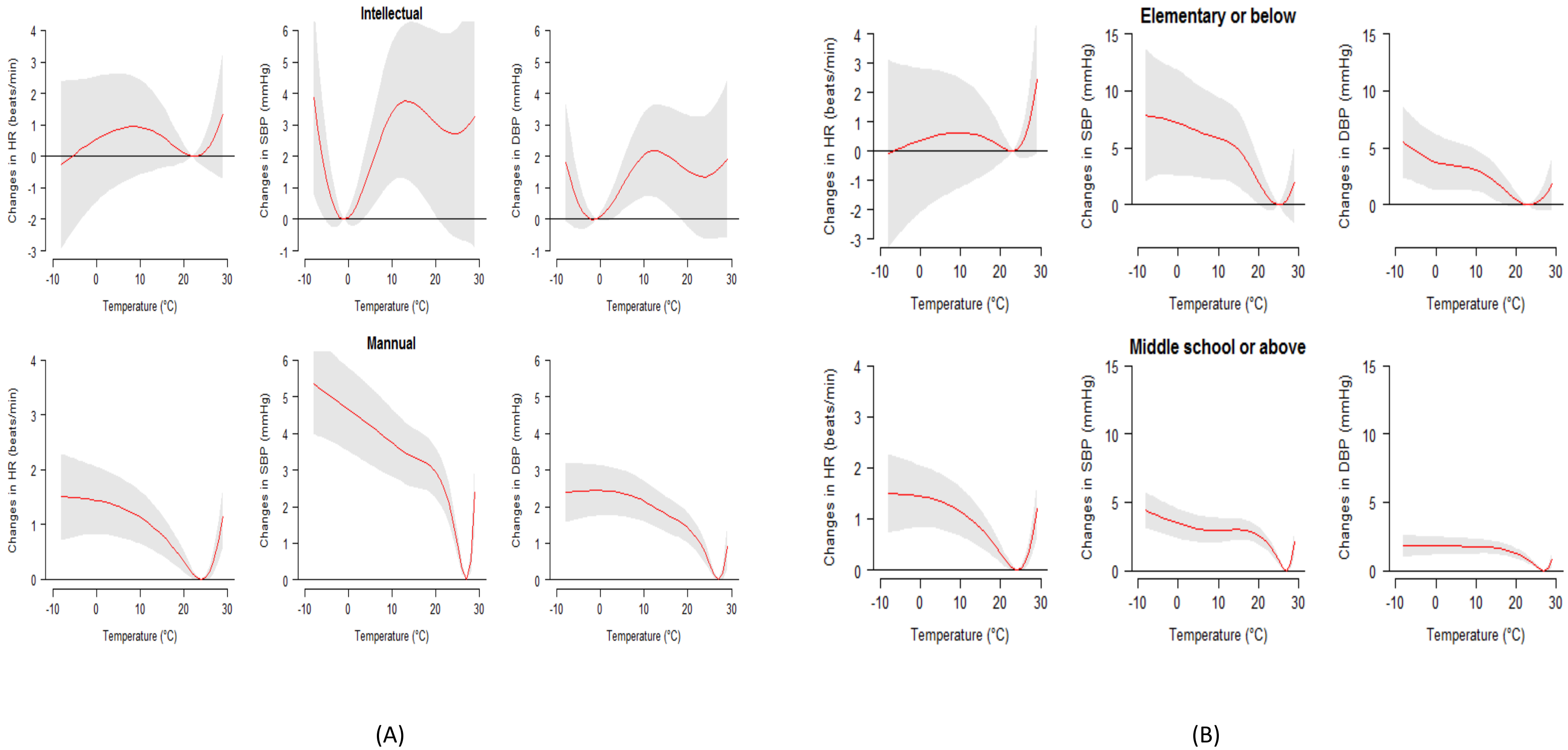

Figure S5. The estimated effects of 3-day moving average of mean temperature on HR, SBP and DBP among people with different work types (A) and different education levels (B)

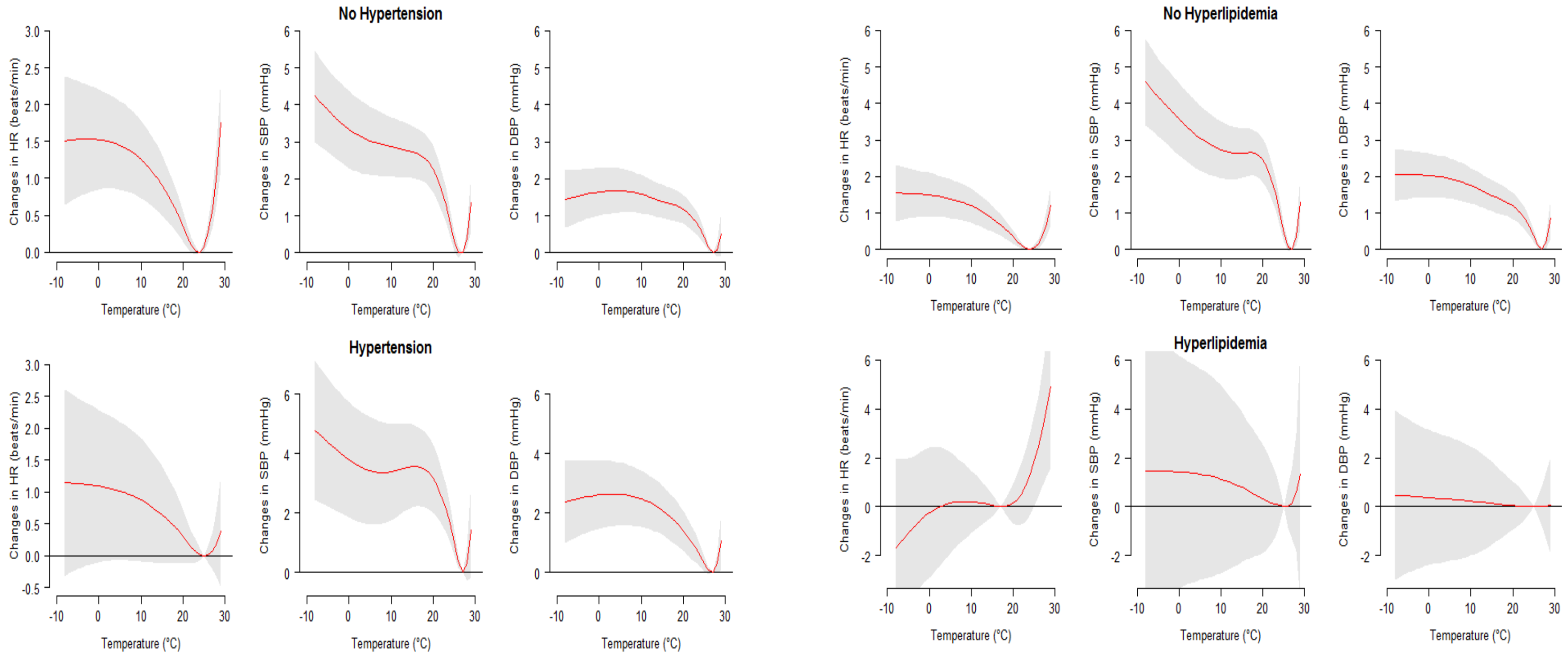

(A) (B)

Figure S6. The estimated effects of 3-day moving average of mean temperature on HR, SBP and DBP among people with and without hypertension (A) and people with and without hyperlipidemia (B)
